# Study protocol TransTAM: Transdiagnostic research into emotional disorders and cognitive-behavioral therapy of the adaptive mind

**DOI:** 10.1186/s12888-024-06108-0

**Published:** 2024-10-05

**Authors:** Andrea Hermann, Christoph Benke, Carlo R. Blecker, Benjamin de Haas, Yifei He, Stefan G. Hofmann, Jona R. Iffland, Johanna Jengert-Stahl, Tilo Kircher, Katrin Leinweber, Marcel Linka, Christoph Mulert, Marie K. Neudert, Ann-Kathrin Noll, Christiane A. Melzig, Winfried Rief, Constantin Rothkopf, Axel Schäfer, Christina V. Schmitter, Verena Schuster, Rudolf Stark, Benjamin Straube, Raphaela I. Zimmer, Lukas Kirchner

**Affiliations:** 1 Department of Psychotherapy and Systems Neuroscience, Justus Liebig University of Giessen, Giessen, Germany; 2 Department of Clinical Psychology, Experimental Psychopathology and Psychotherapy, Philipps University of Marburg, Marburg, Germany; 3 Justus Liebig University of Giessen, Bender Institute of Neuroimaging, Giessen, Germany; 4 Experimental Psychology, Justus Liebig University of Giessen, Giessen, Germany; 5 Department of Psychiatry and Psychotherapy, Philipps University of Marburg, Marburg, Germany; 6 Department of Psychology, Philipps University of Marburg, Marburg, Germany; 7 Center of Psychiatry, Justus Liebig University of Giessen, Giessen, Germany; 8 Department of Clinical Psychology, Philipps University of Marburg, Marburg, Germany; 9 Institute of Psychology, Centre for Cognitive Science, Technical University of Darmstadt, Darmstadt, Germany; 10 Center for Mind, Brain and Behavior (CMBB), Philipps University of Marburg and Justus Liebig University of Giessen, Marburg, Germany

**Keywords:** Therapy response, Therapy outcome prediction, Naturalistic outpatient sample, Neurobiological markers, Transdiagnostic markers, Transdiagnostic symptom dimensions, Longitudinal study, Cognitive behavioral therapy

## Abstract

**Background:**

Emotional disorders such as depression and anxiety disorders share substantial similarities in their etiology and treatment. In recent decades, these commonalities have been increasingly recognized in classification systems and treatment programs crossing diagnostic boundaries.

**Methods:**

To examine the prospective effects of different transdiagnostic markers on relevant treatment outcomes, we plan to track a minimum of *N* = 200 patients with emotional disorders during their routine course of cognitive behavioral therapy at two German outpatient clinics. We will collect a wide range of transdiagnostic markers, ranging from basic perceptual processes and self-report measures to complex behavioral and neurobiological indicators, before entering therapy. Symptoms and psychopathological processes will be recorded before entering therapy, between the 20th and 24th therapy session, and at the end of therapy.

**Discussion:**

Our results could help to identify transdiagnostic markers with high predictive power, but also provide deeper insights into which patient groups with which symptom clusters are less likely to benefit from therapy, and for what reasons.

**Trial Registration:**

The trial was preregistered at the German Clinical Trial Register (DRKS-ID: DRKS00031206; 2023–05-09).

## Background

Rather than separate processes being responsible for the onset and maintenance of individual disorders, common transdiagnostic markers and processes linked to multiple mental disorders have been identified on the cognitive-emotional and biological level [1]. This is reflected in the development of alternative classification systems such as the Research Domain Criteria (RDoC) [2] or Hierarchical Taxonomy of Psychopathology (HiTOP) [3], and overarching disorder concepts [4]. For instance,’emotional disorders‘ describe a concept under which different disorders (e.g., anxiety disorders, unipolar depression) are grouped based on shared mechanisms contributing to their onset and maintenance [5]. Based on these conceptualizations, transdiagnostic psychotherapeutic interventions have been developed that incorporate these transdiagnostic mechanisms within treatment programs [4, 6]. However, studies investigating the relative influence and predictive power of a wide range of transdiagnostic factors over time within a naturalistic outpatient setting are lacking. In the TransTAM study, we aim to fill this gap by predicting symptom reduction using selected transdiagnostic factors covering most RDoC domains and units of analysis that have been identified as particularly relevant in the respective research areas. In the following paragraphs, we briefly introduce the most important factors (for additional constructs, see Table 2).

### Perceptual, cognitive and motor variability

Continuous psychophysics [7] is a recent methodological advance that overcomes the rigid structure of classical psychophysics tasks, which typically involve hundreds of trials with binary decisions. Instead, subjects make continuous behavioral adjustments in response to dynamically changing stimuli [8], e.g., by tracking moving targets with a computer mouse [7], their finger [9], or gaze [10]. Such tasks are not only more natural and less tedious for the subjects, but they enable us to characterize properties of the perception–action loop beyond perceptual sensitivity (e.g., numerosity, speed, and contrast) using just a few minutes of data. A recently developed computational modeling framework for continuous psychophysics based on inverse optimal control [11] allows us to infer subjects' motor variability, internal behavioral costs, and subjective beliefs about the stimulus dynamics in a theory-driven fashion. Such quantities have been linked to anxiety and affective disorders: For example, anxiety is related to differences in perceptual sensitivity to discriminating fearful and neutral faces [12, 13]. State anxiety is associated with reduced motor variability, marked by more repetitive and rigid movements [14, 15]. In terms of behavioral costs, the subjective cost of physical effort is higher for patients with major depressive disorder compared to healthy individuals [16, 17]. Despite these clinically relevant findings, less is known about the transdiagnostic relevance of these processes as well as their importance in predicting psychotherapeutic treatment outcome.

### Active vision

The free viewing of natural scenes yields rich data at minimum demand to the observer. It provides insight into general cognitive factors and mechanisms of potential transdiagnostic relevance, such as the individual tendency for visual exploration and social salience [18–20]. Recent research has shown that basic aspects of visual exploration behavior, like saccadic frequency and amplitudes, are diminished under acute fear [21] and in patients with affective disorders [22–25]. Moreover, social anxiety can lead to the visual avoidance of socially significant stimuli like faces and eyes [26]. Finally, affective disorders have been shown to accompany increased reaction times and slower saccadic velocity in pro- and anti-saccade tasks [25, 27]. Despite the high diagnostic potential of active vision, studies investigating its transdiagnostic and prognostic relevance remain scarce.

### Social contextual cues

Social context (e.g., facing a communication partner or seeing a speaker from a lateral perspective) plays a crucial role in communication and mental health, shaping the interpretation and response to language and social cues [28–31]. In mental disorders such as major depressive disorder [32], social anxiety disorder [33, 34], or schizophrenia [35], the ability to accurately interpret and respond to social contextual cues can be impaired, leading to difficulties in social communication and stronger feelings of isolation. Investigating these social factors in a transdiagnostic sample will help to understand the challenges faced by individuals with mental health conditions such as major depressive disorder and anxiety disorders, and highlights the need for interventions that enhance social functioning and reduce misinterpretations in social contexts [36]. The processing of social contextual information while communicating can be investigated by manipulating contextual factors such as gestures and body orientation in video clips [32]. For example, Suffel et al. [32] found that patients with major depressive disorder required increased neural effort, particularly in regions like the left inferior frontal gyrus and anterior cingulate gyrus, to interpret social contextual cues, which may contribute to their social difficulties. Whether the processing of socially relevant contextual cues is of transdiagnostic importance and a relevant predictor of symptom reduction in response to psychotherapy remains unknown.

### Social decision-making

Patients with depressive or anxiety disorders reveal marked distortions in social decision-making during social exchanges. These distortions relate to transdiagnostically relevant problems, such as the lack of reciprocity, social avoidance and mistrust, lack of perspective taking, insensitivity to social rewards, distorted social perception, interpersonal rumination, and pessimistic social expectations (for reviews, see [37–41]). Socioeconomic games such as trust games can be used to examine social decision-making across mental disorders and for behavioral phenotyping [42–44]. Unfortunately, little is known about whether and how patients’ biases in social decision-making during trust games (or other socioeconomic games) predict symptom reduction and other relevant treatment outcomes. It is also unclear whether these biases can be used as transdiagnostic markers of psychopathology in social symptom domains.

### Defensive reactivity

Various emotional disorders have been linked to abnormalities in the processing of and response to bodily sensations [45], which can manifest as dysfunctional defensive mobilization at both neural and behavioral levels [46–49]. Overexpressed defensive mobilization, which is clinically characterized by maladaptive anxiety, fear, and related behavioral changes such as avoidance, is transdiagnostically relevant and constitutes a core feature of several mental disorders [50]. Specifically, anxiety and fear of bodily sensations are known to be closely related to the development, persistence, and treatment-related amelioration of psychopathology [51–53]. To specifically probe potential dysfunctions of defensive circuits in the brain tied to somatic symptoms, previous studies have used interoceptive challenges to induce interoceptive perturbations (e.g., cardiorespiratory symptoms via hyperventilation) [54–57]. Initial evidence from studies with individuals who fear body symptoms revealed a pattern of increased defensive mobilization while anticipating and confronting interoceptive perturbations via hyperventilation [56, 58]. In particular, in patients with panic disorder, defensive mobilization toward interoceptive perturbations co-varied with disorder-specific symptom dimensions [59] and persisted in those patients whose symptoms failed to resolve enough after cognitive behavioral therapy (CBT) [46]. However, existing data on defensive mobilization to somatic symptoms are limited to specific mental disorders as classified by current classification systems of mental disorders (e.g., panic disorder). Our understanding of transdiagnostic processes related to aberrant defensive responding to somatic sensations and their underlying pathophysiological mechanisms is thus limited so far, especially in relation to treatment response.

### Pattern separation

Pattern separation is a hippocampus-dependent mnemonic process that enables the discrimination of similar experiences by forming distinct representations of stimulus features (memory encoding) which are later retrievable from memory [60–62]. Pattern separation extracts the difference in an input stimulus (e.g., today’s parking space) with already stored stimuli (yesterday’s parking space in the same garage) despite overlapping or similar features between the stimuli [63]. Impaired pattern separation is discussed as a risk or maintenance factor for emotional disorders and anxiety disorders in particular [64, 65] because it impacts how individuals process, encode and store information from their surroundings. Pattern separation is associated with fear conditioning and fear overgeneralization [66–68], etiologically relevant processes for emotional disorders [69–71]. The mnemonic similarity task (MST) [72] is used as a measure to assess behavioral pattern separation ability. Bernstein et al. [73] report reduced performance in pattern separation but not general recognition memory in patients with posttraumatic stress disorder and other mental disorders. There is also some evidence that individuals with higher levels of depression are worse at separating patterns than those with lower levels of depression [74], but opposite effects were reported in the discrimination of negative stimuli [75]. These findings emphasize the transdiagnostic relevance of behavioral pattern separation, but we still do not know if it also predicts response to psychotherapy.

### Fear conditioning and generalization

Structural and functional abnormalities in the hippocampus are known to be associated with emotional disorders (e.g., anxiety-related and depressive disorders) [76, 77]. The hippocampus is a key structure in regulating the context-dependent modulation of conditioned fear, e.g., extinction recall in a safe extinction context, as well as the renewal of conditioned fear in a novel and potentially dangerous context [78–80]. Moreover, previous research indicates altered context-dependent extinction recall (e.g., reduced activation of the ventromedial prefrontal cortex) in subjects with PTSD and several anxiety disorders [81–83]. A further relevant mechanism in emotional disorders is fear generalization, namely the transfer of a conditioned fear response to stimuli sharing similarity with a conditioned cue (CS) [84]. If a stimulus resembles the CS + (conditioned cue previously paired with the aversive consequence [unconditioned stimulus]), the hippocampus is thought to initiate a fear response by activating fear-expressing brain regions as in the amygdala [85]. Fear generalization is also of potential transdiagnostic relevance and thereby probably contributes to the development and maintenance of anxiety and stress-related disorders [86–88]. Moreover, there is initial evidence that fear conditioning and generalization are associated with therapy outcome [89–91]. However, previous research leaves open the question whether context-dependent fear conditioning and generalization are related to transdiagnostic symptom dimensions in emotional disorders and can predict CBT-related symptom reduction.

### Emotional facial expression processing

The Hariri Task, also known as the emotional faces task, is a functional magnetic resonance imaging (fMRI) task used to study brain responses to emotional stimuli [92, 93]. Participants view faces expressing strong emotions (like fear or anger) and neutral objects (like shapes or spheres) and are asked to identify if the left or right stimulus is identical to the central one. This task has served to investigate amygdala functioning in conditions like depression and anxiety, where emotional regulation may be impaired. The emotional face-matching task used in fMRI studies has been explored further for its potential to predict treatment outcomes. However, recent research suggests caution in overestimating the predictive capabilities of fMRI, including tasks like the Hariri Task [94]. However, as of now, the predictive power of the Hariri Task and similar fMRI tasks for treatment outcomes remains an ongoing research and development area. The frequent use of the Hariri Task helps to connect findings to other cohorts (e.g., [93]) and to share data efficiently. New analyses approaches with the face-matching task considering for example the repetition of stimuli, neural variability (e.g., [95]) or different emotions (fear vs. anger; e.g., [96]) are promising new avenues to make these tasks more reliable and increase their predictive power in a transdiagnostic sample.

### Emotion regulation

Cognitive restructuring of mental distortions, e.g., via cognitive reappraisal, supposedly plays a crucial role in CBT [97, 98], the gold standard intervention for mental disorders [99, 100]. Thereby, cognitive reappraisal has shown to be impaired across mental disorders as indicated by deviant brain activation during reappraisal when compared to healthy controls (for a meta-analysis see [101, 102]). Recent findings show that especially those brain regions involved in cognitive and emotional processing such as the dorsomedial prefrontal cortex or anterior cingulate cortex predict CBT outcome across different emotional paradigms in anxiety-related disorders [103]. Regarding emotional reactivity and reappraisal, this was established for PTSD [104, 105], social anxiety disorder [106–108], and panic disorder [109, 110]. Similar regions were also identified for predicting therapy response in depression during emotion regulation [111]. Furthermore, brain activation during reappraisal (vs. looking at aversive pictures) outperformed the prediction of therapy response in social anxiety disorder compared to demographic data or symptom severity before treatment [108]. Despite its high transdiagnostic relevance, no study has investigated the prediction of therapy response during cognitive reappraisal in a mixed patient sample suffering from emotional disorders.

### Brain structural connectivity

Structural connectivity anomalies are a consistent finding in patients with unipolar or bipolar depression and anxiety-related disorders (e.g., [112, 113]). Specific transdiagnostic relationships between altered circuits (e.g., for amygdala structural connectivity) have been described. Evidence of alterations in the amygdala’s structural connectivity as visualized in Diffusion Tensor Imaging (DTI) have also been related to the prediction of treatment response [114]. Moreover, inflammation biomarkers such as cytokines are reliably elevated in a subset of patients with unipolar or bipolar depression and anxiety-related disorders, and have been associated with differential treatment responses and poor clinical outcomes. A growing body of literature also describes higher levels of endogenous inflammatory markers and altered, typically lower functional or structural connectivity within these circuits in association with transdiagnostic symptoms such as anhedonia and anxiety in psychiatric populations [115]. Interestingly, findings across neuroimaging modalities have consistently shown that the exogenous administration of cytokines or inflammatory stimuli that induce cytokines disrupts circuits and networks involved in threat detection, anxiety, and interoceptive and emotional processing [115]. Free-water imaging is a model-based approach [116, 117] that augments the DTI model by including a second compartment that accounts for the contribution of free-water throughout the brain; Free-water imaging allows us to disentangle two separate pathologies: one affecting the cellular domain such as axonal degeneration, and a second, more extensive pathology affecting the extracellular domain, potentially neuroinflammation. We recently demonstrated in a pilot study of patients with major depressive disorder [118] increased peripheral inflammatory markers (IL-8/ IL-10 ratio), as well as a positive correlation between the inflammatory profile and average free-water values. Moreover, responders to ketamine treatment showed higher baseline Fractional Anisotropy (FA) in the cellular/tissue compartment of the left cingulum bundle. It is important to mention that with advances in the acquisition of DTI data (multi-band, multi-shell approach) two compartment analyses have been substantially improved and the free water imaging-model fit becomes more robust [119].

### Brain functional connectivity

Resting-state fMRI (rs-fMRI) has become a valuable tool in neuroscience for studying the brain's functional organization, especially in populations that may have trouble performing tasks, ie, infants, elderly individuals, or patients with mental disorders. It offers potential insights into the underlying maladaptive mechanisms of these disorders and opens avenues for developing new diagnostic and therapeutic strategies. For example, patients with major depressive disorder exhibit altered brain dynamics compared to healthy controls, characterized by higher fractional occupancy and temporal stability in specific brain states, particularly one with weaker connectivity within and between all brain networks but higher activity in somatosensory, salience, and attention networks [120]. Research in major depression has also shown that individualized fMRI connectivity patterns before treatment can define signatures of antidepressant and placebo responses [121], the TMS response [122], or ECT response [123]. This suggests that rs-fMRI might help in identifying which patients will likely benefit from specific antidepressant treatments, thus paving the way for more personalized treatment approaches in major depression. Furthermore, resting state neuroimaging data proved to be informative in earlier studies for predictive models in social anxiety disorders with 81% accuracy, 84% sensitivity and 78% specificity [124]. However, rs-fMRI in a more recent study was not a significant predictor of CBT outcome in two large multi-site samples [125], suggesting that a combined analyses in context of other functional assessments in a transdiagnostic sample (with patients with anxiety disorders and depression) is an important avenue to understand the predictive value of relevant features from rs-fMRI. Further research is needed to refine these predictive models and validate their effectiveness in clinical practice.

### Objectives

The abovementioned transdiagnostic factors and processes have so far usually been investigated separately, if at all, or regarding specific disorders. The TransTAM study therefore aims to include and investigate a broad range of transdiagnostic mechanisms potentially relevant for predicting symptom reduction integratively in a mixed patient sample with emotional disorders (anxiety (-related) and depressive disorders) undergoing CBT in a routine outpatient setting. The main objectives of the TransTAM study are to investigate (1) the association between transdiagnostic symptom dimensions and transdiagnostic factors and mechanisms, and (2) the prediction of symptom reduction from various transdiagnostic processes (and their relative importance) across emotional disorders. Due to the broad scope of the measures included in the TransTAM study, a variety of research questions and hypotheses will be investigated. Examples for research questions are listed below:

1. Are transdiagnostic symptom dimensions related to subjective and physiological correlates of defensive mobilization while anticipating and confronting hyperventilation-induced body symptoms?
2. To what extent do indices of social decision behavior during social exchanges (e.g., cooperative behavior, recognizing changes in the social context) predict treatment response in terms of reducing symptom severity and interpersonal problems?

## Methods

### Study design and procedure

This bi-centric prospective-longitudinal observational study is investigating adult patients with emotional disorders receiving routine CBT treatment in one of the two outpatient units of the universities of Giessen and Marburg, Germany. During the probatory phase preceding psychotherapeutic treatment, patients are asked to participate in the TransTAM project if they report having psychological problems related to the disorders specified below and fulfill no exclusion criteria (see below). Structured diagnostic interviews [126–128] will be conducted during routine diagnostics within the probatory phase, and might subsequently lead to the exclusion of participants if any exclusion criteria are fulfilled, for example. Depending on specific exclusion criteria, patients can participate in different study parts: study part 1 (behavioral tasks and questionnaires), study part 2 (peripheral physiological experiment), and study part 3 (MRI measures and experiments). See Table 1 for our inclusion and exclusion criteria.

**Table 1:** Inclusion and exclusion criteria

Inclusion criteria for all participants
1. Currently fulfilling the diagnostic criteria for a diagnosis in sections F32—F39 or F4 of ICD-10
2. Currently seeking cognitive-behavioral psychotherapeutic treatment in one of our two outpatient clinics
3. Therapy duration of at least 12 sessions or a regular end of therapy before 12 therapy sessions must be reached
4. Willingness to participate in the study
Exclusion criteria for all participants
1. Diagnosis from section F2 (according to ICD-10; e. g. schizophrenia)
2. Acute manic/hypomanic episode (diagnosis from domains F30 and F31 according to ICD-10)
3. Acute suicidality
4. Psychotic symptoms (e. g. during a major depressive episode)
5. Organic mental disorders and neurological diseases (e. g. dementia, epilepsy, stroke, multiple sclerosis)
6. Age < 18 years
7. Insufficient knowledge of the German language
8. Significant, not correctable impairment of hearing and/or vision
Additional exclusion criteria for study part 2
1. Cardiovascular or respiratory diseases (e. g. condition following a myocardial infarction, hypertension or hypertension requiring treatment, asthma, chronic obstructive pulmonary disease)
2. Neurodermatitis on the palms of the hands
3. Current severe hearing loss
4. Difficulty breathing during simple physical activities (e. g. walking)
5. Pregnancy
Additional exclusion criteria for study part 3
Standard MRI exclusion criteria (e. g. metallic implants)

### Study population

This study investigates patients fulfilling the diagnostic criteria for any mental disorder from the sections F32—F39 or F4 according to ICD-10 [129] (for exceptions, see Table 1) seeking treatment in one of the two CBT outpatient units of the universities of Giessen and Marburg, Germany. See Table 1 for inclusion and exclusion criteria. The target sample size for the behavioral study part 1 is a minimum of *N* = 200 participants and for study part 2 and 3 *N* = 120 participants. By July 2024, *N* = 134 patients had taken part in study part 1, *N* = 78 patients in study part 2, and *N* = 27 patients in study part 3. This study is conducted in accordance with the Declaration of Helsinki and was approved by the local ethics review boards of the universities of Giessen (2022–0034) and Marburg (2023-24 k, 24–178-BO). The trial was preregistered at the German clinical trial register (DRKS-ID: DRKS00031206; 2023–05-09).

### Measures

#### Interviews and self-report measures (study part 1

Information on sociodemographic variables such as age, gender, relationship status, educational and occupational attainment, as well as anamnestic information on past and present medication, drug intake, and treatment is being collected through self-reporting.

The *primary outcome* is defined as the change score in the GSI (Global Severity Index) of the Brief Symptom Inventory (BSI) [130] measured during baseline (probatory phase) and between therapy sessions 20–24, or at the end of therapy if reached earlier. *Secondary Outcomes* and additional self-report questionnaires for basic research questions (baseline assessment) are presented in Table 2 [131–159]. Besides data collection before initiating therapy (probatory phase), primary and secondary outcomes will be collected again at sessions 20—24 and at each participant’s end of therapy. In addition to predictive questions, our project will also address other research questions (e.g., main effects of experiments and paradigms investigating transdiagnostic mechanisms and factors, and their association with psychopathology and transdiagnostic symptom dimensions).

**Table 2:** Self-report questionnaires and therapist ratings

Measure	Description
**Primary outcome**	
Brief Symptom Inventory (BSI) [130]	Psychological distress
**Secondary outcomes**	
Affective Styles Questionnaire (ASQ) [157]	Affective Styles
Anxiety Sensitivity Index-3 (ASI-3) [142]	Anxiety sensitivity
Beck Depression Inventory II (BDI-II) [138]	Depression
Behavioral Inhibition Scale/ Behavioral Activation Scale (BIS/BAS) [154]	Inhibition and avoidance
Big Five Inventory (BFI-10) [149]	Personality
Clinical Global Impression (CGI) [137]	Symptom severity and treatment response
Disability Index (DI, adapted version of the Pain Disability Index) [146]	Impairments, focusing on physical and mental complaints
Fear of Negative Evaluation Scale (SANB-5) [141]	Fear of negative evaluation
Generic Rating Scale for Previous Treatment Experiences, Treatment Expectations, and Treatment Effects (GEEE) [150]	Therapy expectations
Heidelberg Form for Emotion Regulation Strategies (HFERST) [140]	Emotion regulation
Interpersonal Emotion Regulation Questionnaire (IERQ) [139]	Interpersonal emotion regulation
Interpersonal Trust Short Scale (KUSIV-3) [132]	Interpersonal trust
Intolerance of Uncertainty Scale (IUS) [136]	Intolerance of uncertainty
Inventory of Interpersonal Problems (IIP-D-32) [156]	Interpersonal problems
Multidimensional Emotional Disorder Inventory (MEDI) [151]	Transdiagnostic symptom dimensions
Patient-Reported Outcomes Measurement Information System Level 2 (PROMIS-Level 2) [155]	Anxiety
Penn State Worry Questionnaire (PSWQ) [153]	Worrying
Perseverative Thinking Questionnaire (PTQ) [135]	Perseverative thinking
Process-Based Assessment Tool (PBAT) [134]	The process of change in therapy
Response Styles Questionnaire (RSQ-K) [144]	Rumination
Social and Occupational Functional Assessment Scale (SOFAS) [147]	Social and occupational functioning
State-Trait Anxiety Inventory, Trait-Anxiety (STAI-T) [145]	Trait anxiety
Temporal Experience of Pleasure Scale (TEPS) [152]	Anticipatory and temporal pleasure
WHO-5 [133]	Well-being and mental health
**Further questionnaires (baseline assessment)**	
Brief Assessment of Gesture (BAG) [148]^a^	Gestures
Childhood Trauma Questionnaire (CTQ) [159]	Traumatic childhood experiences
Emotion Regulation Questionnaire (ERQ) [131]^a^	Habitual emotion regulation
Life Events Checklist for DSM-5 (LEC-5) [158]	Traumatic experiences
Posttraumatic Stress Disorder Checklist for DSM-5 (PCL-5) [143]	Traumatic experiences and trauma-associated symptoms

^a^Assessment only during study part 3

#### Behavioral tasks (study part 1)

##### Continuous psychophysics task

A recently developed experimental paradigm called “continuous psychophysics” abandons the rigid structure imposed by standard psychophysical tasks such as the two-alternative forced choice paradigm, and instead elicits continuous behavioral adjustments to dynamic stimuli [7]. While invaluable, the classic paradigms consisting of a succession of hundreds of trials in which stimuli are presented briefly and a participant responds with a binary decision, lead to participants’ low engagement levels, particularly in untrained subjects, resulting in measurements contaminated by additional variability. Instead, continuous psychophysics enables us to collect behavioral data much faster and the task itself, e.g., the employed manual tracking task, has been described by participants as being engaging and fun. In the current task, subjects manually track with their fingers Gaussian blobs of different luminance contrast embedded in a white noise background shown on a monitor [7]. Individual trials take 30 s and a total of 5 min worth of continuous psychophysics data, allowing us to infer the perceptual thresholds as about 1,5 h of two-alternative-forced-choice task's data. With a new analysis method [11] it is also possible to quantify not only individual participants' perceptual uncertainty, but also their motor variability, the effort cost of carrying out the task relative to the movement costs, and properties of participants' internal model of the target motion together with its uncertainty.

##### Natural viewing task

Here, we will employ a recently developed free-viewing paradigm that yields robust estimates of individual exploration tendencies and social salience using just 40 images [18, 20]. We will quantify a range of gaze features and probe their covariation with symptoms and their relief across therapy. Specifically, we will probe saccade frequency, median saccadic amplitude and the number of objects fixated as markers of the individual tendency for visual exploration (cf. [160]). We will furthermore quantify the proportion of first fixations and dwell time falling on faces, eyes and persons as markers of individual social salience. Last of all, we will probe saccadic latencies and error rates during a gamified pro- and anti-saccade task.

##### Mnemonic discrimination task (MST)

This performance will be assessed with the MST for objects [72], including an encoding and test phase. During the encoding phase, 128 pictures of everyday objects are presented for 2 s on a white background on a monitor and should be categorized by participants whether they display an indoor or outdoor item. After a response is given, the next trial starts with an inter-stimulus-interval (ISI) of 500 ms. The test phase of the MST immediately follows the encoding phase and serves as a memory test. During the test phase, 64 pictures of objects presented during the encoding phase (condition: ‘old’), 64 pictures of new objects (condition: ‘foils’), and 64 pictures showing similar but not identical items presented during the encoding phase (condition: ‘lure’) are shown for 2 s in a random order. Participants indicate by button press if the picture they see is an ‘old’, ‘new’, or ‘similar’ item. The next trial starts with an ISI of 500 ms once a response has been given. The ‘Lure Discrimination Index’ will be used as a main indicator for mnemonic discrimination performance (pattern separation). It is composed of the number of similar responses given to lure items (correctly classified lure objects) minus the number of similar responses given to foils to correct for a general bias to respond with “similar”. A higher ‘Lure Discrimination Index’ indicates better identification of similar items (better behavioral pattern separation performance).

##### Social decision-making task

Participants will take part in 60 rounds of a trust game. Each round allows participants to either keep a fixed amount of money or donate it to a “fellow player” (in fact computer-controlled agents). If participants keep the money, it is added to their account, and the next round begins. If they donate, the fellow player may reciprocate with more money (cooperation) or retain it all (exploitation) before the next round starts. Participants will be randomly assigned to one of three experimental conditions. In the ‘positive-to-negative’ condition, they interact with mainly cooperative players for the first 30 rounds, then with mostly exploitative players for the last 30 rounds. The ‘negative-to-positive’ condition reverses this order. In the ‘random’ condition, the fellow player’s behavior is randomized. Participants are led to believe they are playing with real people, with new players each round. In reality, all fellow players are computer-controlled. Participants will receive 50% of their game earnings in addition to a time-based compensation, incentivizing serious participation. The primary focus of this study is to measure participants’ decisions to keep or donate money throughout the rounds.

#### Peripheral physiological experiment (study part 2)

##### Defensive reactivity task

Electromyographic activity over the left musculus *orbicularis oculi*, electrodermal and electrocardiographic activity, as well as respiration will be registered by bioamplifier as reported elsewhere [59, 161]. Data will be acquired via AcqKnowledge software (Cook, 1987) (Marburg) or eegoTM software (Version 1.8.2., eemagine Medical Imaging Solutions GmbH, Berlin, Germany) (Giessen). The laboratory session will start with a 2 min adaptation phase followed by a rating of anxiety and of the severity of the 14 DSM-5 panic symptoms on a Likert Scale ranging from 1 (not at all) to 10 (very strong) via computer keyboard. Then, one-half of the participants will start with the hyperventilation condition, while the other half will start with the control (safe) condition followed by the other condition, respectively. Interoceptive perturbations, that is, bodily sensations, will be elicited using a highly standardized hyperventilation (HV) task. This HV procedure is highly efficient in inducing a variety of bodily sensations [162] that persist for several minutes after the breathing exercise is discontinued [56]. The hyperventilation condition comprises of a 1.5 min anticipation of the HV phase, followed by a 3 min HV task and 5 min resting phase (post-HV phase). The control condition comprises of a 1.5 min safe (no HV) phase, then a 5 min resting phase. Defensive reactivity will be continuously assessed throughout the anticipation and post-HV/resting phase, and retrospective ratings of HV-elicited symptoms will be acquired as described above at the end of this phase. The hyperventilation task will be introduced to the participants as a ‘fast breathing exercise’. During the 3 min HV task, tones of rising and falling pitch will be heard via headphones prompting the participants to breathe at a respiratory rate of 20 cycles/min. To assess compliance with the HV procedure, the respiratory rate (RR) and CO2 of the expired air (p_et_CO2) will be continuously monitored by a Nellcor NPB-70/N85 Capnograph (Nellcor Puritan Bennett, Pleasanton, CA). Visual feedback (instruction slides) will be used to lead the participant to ‘breathe deeper’ until they reach a target p_et_CO2 of 20 mmHg. Using further visual feedback (‘breathe more shallow’, ‘deeper,’ or at a ‘constant depth’), the breathing depth will be adjusted throughout the hyperventilation task to maintain the target p_et_CO_2_. Visual feedback will be given by the experimenter who tracked p_et_CO_2_ levels online. A 50 ms burst of broadband white noise (95 dBA, rise/fall time < 1 ms) will be presented binaurally via AKG K-72 headphones (AKG Acoustics GmbH, Austria) to serve as a startle-eliciting stimulus. Startle probes will be presented using Presentation software. During the adaptation phase, six startle probes will be delivered to habituate startle response magnitudes to a stable baseline (mean inter-probe interval: 15 s; range: 10—20 s). Five startle probes will be presented during the anticipation of HV and safe phase. Fifteen startle probes (three per minute; mean inter-probe interval: 20 s; range: 10—30 s) will be presented during both the 5 min post-HV and control phase, respectively. No startle probes will be presented during the guided breathing task.

#### Magnetic resonance imaging (study part 3)

Magnetic resonance imaging will take place at the Bender Institute of Neuroimaging, Justus Liebig University Giessen (Magnetom Prisma 3T scanner, Siemens Healthineers AG, with a 64 channel Head/Neck coil), and at the Department of Psychiatry and Psychotherapy, Philipps University Marburg (SIGNA™ Premier 3T wide-bore MRI scanner, GE, with a 48-channel-head coil). An anatomical image (T1) will be acquired to normalize functional imaging data and investigate gray matter differences. Moreover, diffusion tensor imaging will be applied to investigate structural connectivity. Resting State functional magnetic resonance imaging will be conducted to assess intrinsic networks in the brain while no task is being performed. Specific MRI sequences regarding the experimental tasks (social context task, emotional faces task, fear generalization task, emotion regulation task) will be detailed in the respective publications. Visual stimuli are presented via presentation software (Neurobehavioral Systems). All stimuli are displayed on a monitor behind the scanner and participants are able to see the monitor via a mirror mounted to the head coil.

##### Social context task

For a complete description of the stimulus set and the evaluation and procedures see previous studies [30, 32]. Twenty German sentences are presented to the participants as short videos. The grammatical structure is consistent across all sentences: subject–predicate–object. Each sentence is presented once with an iconic co-speech gesture and once with no gesture. The co-speech gesture is performed in a natural way, conforming with the content of the sentences, for example, ‘The man caught a big fish,’ while the actor indicates the size of the fish with his hands. For 0.5 s at the beginning and end of each clip, the actor neither speaks nor moves. Two cameras have simultaneously filmed the actor while speaking, so that only the context (viewpoint) differs between the frontal and lateral condition. Four different experimental sets consisting of the same stimuli but in counterbalanced sequential arrangements regarding body orientation and gesture presence were created. One of these sets was selected for TransTAM to reduce between subject variability possibly triggered by task sequence. Each stimulus set consists of 80 video clips in total (40 frontal, 40 lateral conditions). Prior to the fMRI scanning procedure, participants will see and evaluate four practice trials (videos are not part of the main experiment) to make sure they have understood the task. For the fMRI experiment, MRI- compatible headphones together with earplugs will be used to optimize scanner noise reduction. Stimuli will be presented in the middle of the video screen. The 20 videos for each of the four conditions will have been presented in identical pseudorandomized order (one out of four sets applied in a previous study [30]) across subjects to increase comparability between subjects. After the presentation of each video, a low-level baseline with varying duration of 3,750–6,750 ms (mean = 5,000 ms) follows. This baseline consisted of a blank gray screen. A similar experimental procedure was used in earlier studies of ours (for details, see, for example, [30, 31]). For each stimulus, participants are asked to evaluate whether they felt addressed or not, taking into account the entire video. To give their answer, participants are instructed to press a button for ‘yes’ or ‘no’ on a magnetic resonance-compatible response pad. Thus, feeling addressed results in a button press with the right index finger, not feeling addressed results in a right middle finger button press. Participants are further instructed to respond immediately once the video has disappeared from the screen. For statistical analysis, the ratings (number of yes responses for the 20 videos per condition) will be transformed into percentage of ‘yes’ responses related to all responses of one condition for each subject and condition.

##### Emotional faces task: Face-matching paradigm

This paradigm is widely used in the imaging genetics field for robustly eliciting amygdala responses to fearful and angry faces. The paradigm has been employed in numerous imaging studies investigating amygdala responsiveness, including our own group’s (e.g., [92, 93]). The experimental task includes four face-processing blocks interleaved with five sensorimotor control blocks (e.g., [163, 164]). In the face-processing block participants view one face in the upper half of the screen and two faces in the bottom half of the screen. Participants will be asked to identify which of the two bottom faces matched the upper face. Each face-processing block will contain a different set of six matching images of a single emotional facial expression (anger, fear, or neutral). Participants will be randomly assigned to one of four block orders. Facial expression images consist of a subset of stimuli from the pictures of facial affect set [165], balanced for gender. In the sensorimotor control blocks, participants view a geometric shape (vertical ellipses or horizontal ellipses) in the upper half of the screen and two shapes in the bottom half of the screen. All blocks are preceded by brief instructions (‘Match faces’ or ‘Match shapes’) lasting 2 s. In the face processing blocks, each face trio will be presented for 4 s with a variable interstimulus interval of 2–6 s (mean = 4 s) for a total block length of 48 s [164]. A variable interstimulus interval will be used to minimize expectation effects and resulting habituation and to maximize amygdala reactivity throughout the paradigm. In the sensorimotor control blocks, each shape trio will be presented for 4 s with a fixed inter-stimulus interval of 2 s. Subject performance (accuracy and reaction time) will be monitored during all scans.

##### Fear conditioning and generalization task

We have extended the context-dependent fear conditioning paradigm used in previous studies [78, 80, 81] by adding a fear generalization and delayed extinction generalization phase. Fear acquisition and subsequent fear generalization take place in context A, and extinction training in context B. One week (6 -8 days) later, extinction recall is performed in context B and fear renewal in a novel context C. Pictures of different rooms (office room, conference room, room with a shelf) constitute contexts A, B and C. Each room contains the same initially turned off desk lamp. During the experiment, the desk lamp lights up in blue and green. The light colors serve as conditioned cues (CS). One light color (CS +) is sometimes followed by electrical stimulation (2 ms pulses with 200 Hz for a duration of 500 ms, unconditioned stimulus, UCS) during the acquisition phase (62.5%) and during the fear generalization phase (50%). The other light color (CS-) is never followed by electric stimulation. Electrical stimulation (UCS) is delivered through electrodes (Digitimer DS7A, Digitimer Ltd, UK) attached to the inside of the right forearm, a thumb-width below the thenar. The intensity of electric stimulation is adjusted before the experiment for each participant to a level perceived as unpleasant but not painful. Both CS (CS + , CS-) are presented during all experimental phases. Three other light colors, which were located in the Derrington-Krauskopf-Lennie (DKL) space between both CS, serve as generalization stimuli (GS). The GS shows a difference of 16 degrees in the DKL space to the stimulus most similar to them (CS + , GS_1_, GS_2_, GS_3_, CS-). GS_1_ is most similar and GS_3_ least similar to the CS + ; GS_3_ is most similar and GS_1_ least similar to the CS-; GS_2_ is exactly between CS + and CS- in terms of color (50% similar to CS + and CS-). The three different GS are presented in addition to the conditioned cues during the fear generalization phase on study day 1 and during the extinction recall and fear renewal phases on study day 2. Each trial starts with a white fixation cross on a black background (jittered between 625 and 2,500 ms). Then, the context picture is presented for 3 s, showing a turned off desk lamp. After 3 s, the context picture is presented with a desk lamp which lights up in one color for 6 s. Next, a white fixation cross on a black background appears on screen for a total trial duration of 20 s. The trial structure is identical over all phases except for the fear acquisition and fear generalization phases. During the fear acquisition phase, the CS + is followed by electric stimulation after CS + offset in 62.5% of the cases. During the fear generalization phase, the CS + is followed by electric stimulation after CS + offset in 50% of the cases. Fear acquisition in context A consists of 16 trials (8 trials per CS), fear generalization in context A on day 1 (as well as extinction recall in context B and fear renewal in context C on day 2) each comprise 40 trials (8 trials per CS and GS). The trials are arranged in two blocks during all experimental phases. Each block comprises half of the CS + and CS- (and GS) trials presented in pseudorandomized order (no more than twice the same condition after another). The first two and last two trials of each phase consist of one CS + and one CS-, respectively. During fear acquisition, the first CS + is always reinforced to promote fear learning. In addition, the last CS + is reinforced to avoid premature extinction learning during fear acquisition. Participants are informed about the trial structure and content before fear acquisition: They are instructed to attentively watch the presentation showing a room with an initially turned off desk lamp, which will light up in one color after a few seconds. They are then instructed about the possibility to receive electrical stimulation at the end of the presentation of the turned-on lamp. They are informed that electrical stimulation will sometimes follow one light color, but not another light color. For all other experimental phases, they are instructed that the next experimental phase will resemble the previous one and that they may or may not receive electrical stimulation. Besides blood oxygen level-dependent responses in regions of interest, electrodermal responses will be measured during the task, and ratings regarding the CSs and contexts will be assessed after the last experimental phase.

##### Emotion regulation task

Participants carry out an adapted version of an emotion regulation task [166] during fMRI. They are instructed to either watch aversive (‘Look negative’) or neutral (‘Look neutral’) pictures from the International Affective Picture System (IAPS; [167]) or to reappraise negative pictures (‘Reappraisal’). In the reappraisal condition, participants are instructed to reduce the intensity of their negative feelings by imagining a better ending of the situation or that the situation is better than indicated. During the’look’ conditions, participants should simply watch the depicted scenarios without actively changing their emotional state evoked by the pictures. In a pseudorandomized block design, each block starts with a jittered presentation of the regulation instruction (i.e. ‘Look’ or ‘Reappraise’), which is followed by the presentation of 4 negative or 4 neutral pictures (each picture is presented for 5 s without an interstimulus interval) according to the presented condition. After each block, participants are asked to rate the intensity of currently perceived negative feelings on a 7-point Likert scale (1 = no negative feelings at all; 7 = very strong negative feelings) via a button press (max 5 s). A white fixation cross on black background is then presented during the inter-trial-interval up to a total block duration of 30 s. The total task consists of four blocks per condition (12 blocks in total) and lasts for 6 min 37 s. The 12 blocks are arranged in four runs with a randomized presentation of all conditions within each run, leading to a maximum of two presentations of the same condition in succession.

### Proposed statistical analyses

To evaluate how well the transdiagnostic markers or processes predict symptom reduction, we will take more tailored and more integrative statistical approaches depending on the individual paradigms and additional research questions.

At the paradigm level, we plan to use simple and multiple linear regression models to explore the predictive power of individual predictors on symptom reduction. We may make group comparisons to answer additional research questions (e.g. responder vs. non-responder) or more complex statistical techniques such as hierarchical linear modeling or computational modeling. Correction for multiple comparisons will be applied if necessary (depending on the respective research question/paradigm).

At the integrative level, we will also conduct multiple linear regression analysis or generalized linear mixed models. These models will help us determine how effectively combinations of variables predict different symptom domains and treatment outcomes. We also plan to take network analytical approaches to examine the relationships among (and relative importance of) different markers, symptoms, and outcomes, both cross-sectionally and longitudinally.

### Power calculation

**Fig. 1:**
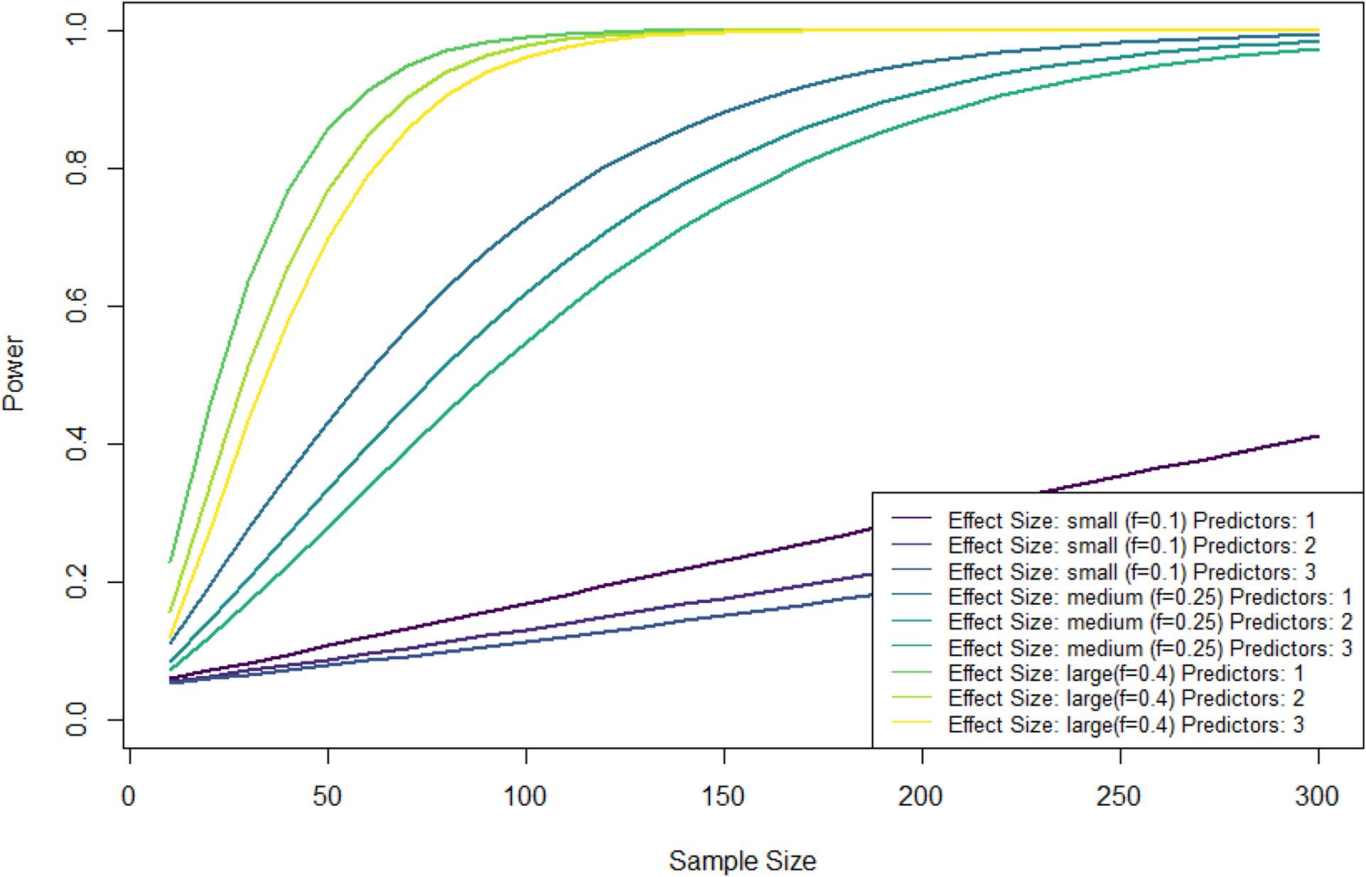
Power analyses for different effect sizes, sample sizes and numbers of predictors

We calculated statistical power for different effect sizes, sample sizes and numbers of predictors assuming simple or multiple linear regression analysis with an alpha level of 0.05. Figure 1 shows that even with substantial dropout, medium effects can still be detected with a statistical power of 80%.

## Discussion

### Aim of the study

The aim of this bi-centric, multimethod prospective-longitudinal observational study is to investigate the association between transdiagnostic factors and mechanisms and 1) transdiagnostic symptom dimensions before psychotherapy and 2) the reduction of these symptoms following CBT in patients with emotional disorders undergoing CBT in a routine outpatient setting. Specifically, we are interested in understanding how maladaptations in crucial psychological domains are associated with transdiagnostic symptom dimensions in emotional disorders probably by having an impact on their development and/or maintenance. Moreover, we aim to figure out how more advantageous adaptation processes might predict CBT response in a naturalistic setting. Does a more adaptive regulation of negative emotions or a stronger generalization of fear extinction (as reflected in stronger ventromedial prefrontal cortex activation) predict stronger symptom reduction in response to CBT? In addition to our focus on single mechanisms, we will take integrative approaches to exploit the complementary variance explained by each task to improve the prediction of treatment outcome.

### Strengths

Covering a broad range of mechanisms comprising perceptual, motoric, mnemonic, social, cognitive and emotional functions is a crucial strength of this study, allowing a combined investigation of relevant factors and their relative contribution to transdiagnostic symptom dimensions and symptom reduction in emotional disorders. This interdisciplinary cooperation including basic and clinical researchers moreover enables us to have different perspectives on relevant predictors for transdiagnostic symptom dimensions and symptom reduction, and to explore new avenues for deeper understanding of mental disorders and their treatment. This bi-center approach enables us to recruit a large number of patients (≈ 200 patients/year), at least for the behavioral study part, in which they receive routine CBT. Furthermore, a great opportunity of this study is that CBT is implemented in a rather ‘naturalistic’ setting comprising treatment by licensed psychotherapists or psychologists in psychotherapy training (under supervision) applying state-of-the-art CBT interventions but in an individual patient-focused manner. We have also included ecologically valid experimental paradigms by considering contextual variables, naturalistic behavior (free viewing task), or ‘real-life’ social interactions. This enables an evaluation and prediction of transdiagnostic symptom correlates and treatment responses closer to reality. In addition, our study’s transdiagnostic approach considers and disentangles various problems associated with disorder-specific clinical research: as comorbidity is the rule rather than the exception in treatment-seeking psychotherapy outpatients, our transdiagnostic and dimensional approach enables the unrestricted inclusion of all patients presenting comorbid disorders (except some uncommon disorders as described above). In addition, this procedure takes into account the dimensional nature of mental disorders by also considering single symptoms (e.g., intrusions) without a full representation of the related clinical diagnosis (e.g., posttraumatic stress disorder). We moreover try to predict symptom reduction by regarding the entire spectrum of emotional problems rather than relying on symptom severity regarding a specific diagnosis. It is also a great advantage of our study that we use multiple methods to capture crucial components of the suggested mechanisms. The applied methods range from behavioral (e.g., continuous psychophysics, eye-tracking, ratings, reaction times) to psychophysiological methods (e.g., electrocardiogram, skin conductance responses, MRI), enabling the investigation of more or less automatic and objective as well as subjective processes.

### Challenges & limitations

Despite these various advantages, our study has also some challenges and limitations. As it is a naturalistic design, all eligible patients (and willing to participate in the study) seeking treatment at one of the outpatient clinics, are included. As a result of this approach, the distribution of specific disorders and the most frequent symptoms in the final sample are relatively unpredictable. This might for example lead to an overrepresentation of depressive disorders or symptoms, which we will however consider in our transdiagnostic analysis approach. In addition, the naturalistic setting precludes treatment according to standardized protocols and thus limits the validity of our findings. Another limitation is the absence of a waitlist control group, although the main question concerns the prediction of symptom reduction rather than the CBT effect. Nevertheless, any changes in symptom measures occurring during the study time period might also be related to factors and influences other than treatment alone. In addition, as symptom reduction at therapy sessions 20–24 is our primary outcome measure, it is not entirely clear how strong symptoms are already reduced at this point.

### Interpretation of data regarding methodological considerations

The interpretation and generalization of our results might be restricted due to a bias in patient selection (only those who are willing to participate and fulfil no exclusion criteria). Despite investigating a treatment-seeking sample, the variance in symptom severity might be restricted as only patients with an indication for out-patient CBT have been included but not those requiring in-patient treatment or refusing any treatment for various reasons. Moreover, a large number of researchers together with the inclusion of several transdiagnostic factors and mechanisms might compromise a unified interpretation of our findings. Beyond our specific research questions and hypotheses, additional exploratory analyses are also possible. The broad range of potential mechanisms and predictors included will compound the overall risk of false positive results. We therefore aim to replicate major findings of interest in a follow-up study with a larger sample size.

## Summary and implications

The TransTAM study is relying on behavioral, peripheral physiological and neuroimaging markers to investigate whether transdiagnostic factors and mechanisms are related to transdiagnostic symptom dimensions and predict symptom reduction in patients with emotional disorders. Our study directs both a transdiagnostic and naturalistic focus by investigating a treatment-seeking patient sample receiving standard CBT in two outpatient clinics under naturalistic conditions. We moreover have aimed to conduct a (as far as possible) realistic investigation of the proposed mechanisms in order to enhance the ecological validity of our findings. New insights into transdiagnostic factors and mechanisms might help to improve existing and develop novel treatment options for emotional disorders.

## Data Availability

n.a.

## Abbreviations

TransTAM: Transdiagnostic Research into Emotional Disorders and Cognitive-Behavioral Therapy of the Adaptive Mind
RDoC: Research Domain Criteria
HiTOP: Hierarchical Taxonomy of Psychopathology
CBT: Cognitive Behavioral Therapy
MST: Mnemonic Similarity Task
fMRI: Functional Magnetic Resonance Imaging
DTI: Diffusion Tensor Imaging
IL-8/ IL-10 ratio: Peripheral inflammatory markers
FA: Fractional Anisotropy
rs-fMRI: Resting-state fMRI
ICD-10: International Classification of Diseases 10th Revision
GSI: Global Severity Index
BSI: Brief Symptom Inventory
CGI: Clinical Global Impression
SOFAS: Social and Occupational Functional Assessment Scale
BDI-II: Beck Depression Inventory II
WHO-5: World Health Organisation-Five Well-Being Index
DI: Disability Index
MEDI: Multidimensional Emotional Disorder Inventory
ASQ: Affective Styles Questionnaire
IERQ: Interpersonal Emotion Regulation Questionnaire
HFERST: Heidelberg Form for Emotion Regulation Strategies
BFI-10: Big Five Inventory
PSWQ: Penn State Worry Questionnaire
RSQ-K: Response Styles Questionnaire
PTQ: Perseverative Thinking Questionnaire
STAI-T: State-Trait Anxiety Inventory, Trait-Anxiety
PROMIS-Level 2: Patient-Reported Outcomes Measurement Information System Level 2
ASI-3: Anxiety Sensitivity Index-3
SANB-5: Fear of Negative Evaluation Scale
IUS: Intolerance of Uncertainty Scale
IIP-D-32: Inventory of Interpersonal Problems
KUSIV-3: Interpersonal Trust Short Scale
BIS/BAS: Behavioral Inhibition Scale/ Behavioral Activation Scale
TEPS: Temporal Experience of Pleasure Scale
PBAT: Process-Based Assessment Tool
GEEE: Generic Rating Scale for Previous Treatment Experiences, Treatment Expectations, and Treatment Effects
CTQ: Childhood Trauma Questionnaire
LEC-5: Life Events Checklist for DSM-5
PCL-5: Posttraumatic Stress Disorder Checklist for DSM-5
DSM-5: Diagnostic and Statistical Manual of Mental Disorders
ERQ: Emotion Regulation Questionnaire
BAG: Brief Assessment of Gestures
ISI: Inter-stimulus-interval
HV: Hyperventilation
RR: Respiratory rate
p_et_CO2: CO2 of the expired air
CS: Conditioned stimulus
UCS: Unconditioned stimulus
GS: Generalization stimuli
IAPS: International Affective Picture System

## Data availability

No datasets were generated or analysed during the current study.
